# Knockdown of Dinoflagellate Cellulose Synthase *CesA1* Resulted in Malformed Intracellular Cellulosic Thecal Plates and Severely Impeded Cyst-to-Swarmer Transition

**DOI:** 10.3389/fmicb.2019.00546

**Published:** 2019-03-19

**Authors:** Wai Sun Chan, Alvin Chun Man Kwok, Joseph Tin Yum Wong

**Affiliations:** Division of Life Science, The Hong Kong University of Science and Technology, Hong Kong, Hong Kong

**Keywords:** cellulose synthesis, CesA, dinoflagellate, cell wall, cellulose synthase, thecal plates, cyst

## Abstract

Cellulose synthesis (CS) is conducted by membrane-bound cellulose synthase complexes (CSCs), containing cellulose synthases (CesA), that are either arranged in hexagonal structures in higher plants or in linear arrays in most microbial organisms, including dinoflagellates. Dinoflagellates are a major phytoplankton group having linear-type CSCs and internal cellulosic thecal plates (CTPs) in large cortical vesicles. Immunological study suggested CesA1p were cortically localized to the periphery of CTPs. During cyst-to-swarmer transition (T_C–S_), synchronized peaks of *CesA1* transcription, CesA1p expression, CS and CTP formation occurred in respective order, over 12–16 h, strategically allowing the study of CS regulation and CTP biogenesis. *CesA1*-knockdown resulted in 40% reduction in CesA1p level and time required for swarmer cells reappearance. CTPs were severely malformed with reduced cellulose content. As CTPs are deposited in internal organelle, the present study demonstrated dinoflagellate CesA1 ortholog was adapted for non-surface deposition; this is different to paradigm of other CesAps which require plasmamembrane for cellulose fiber deposition. This pioneer gene-knockdown study demonstrated the requirement of a gene for dinoflagellate cell wall remodeling and proper T_C–S_, which are prominent in dinoflagellate life-cycles.

## Introduction

Cellulose, the most abundant biopolymer on earth, is the major cell wall polysaccharide component in plants, protists, and alga (Niklas, 2004). Cellulose synthesis (CS) is conducted at the plasma membrane by cellulose synthase (CesA) complexes (CSCs), containing CesA subunits. CSCs are organized either as rosette-like structures in higher plants and green algae, or as different linear multimeric structures in prokaryotes, protists, tunicates, and other algae (Grimson et al., 1996; Kimura and Itoh, 1996; Domozych, 2016). Biosynthesis of crystalline cellulose involves polymerization of glucose and simultaneous aggregation of the resulting glucan chains (Benziman et al., 1980). Apart from the conserved glycosyltransferase catalytic center and the overall arrangement of multiple transmembrane domains, there were little common sequence motifs shared among CesAs from different cellulosic lineages (Roberts and Roberts, 2007), implicating the involvement of different mechanisms for CesAp assembly and post-polymerization processing of glucan chains. Despite linear-type CSCs are commonly deployed among most cellulosic eukaryotic taxons (Brown, 1990; Itoh, 1990; Kimura and Itoh, 1996), little is known about the molecular mechanisms involved in the CS by linear-type CSCs.

**FIGURE 1:**
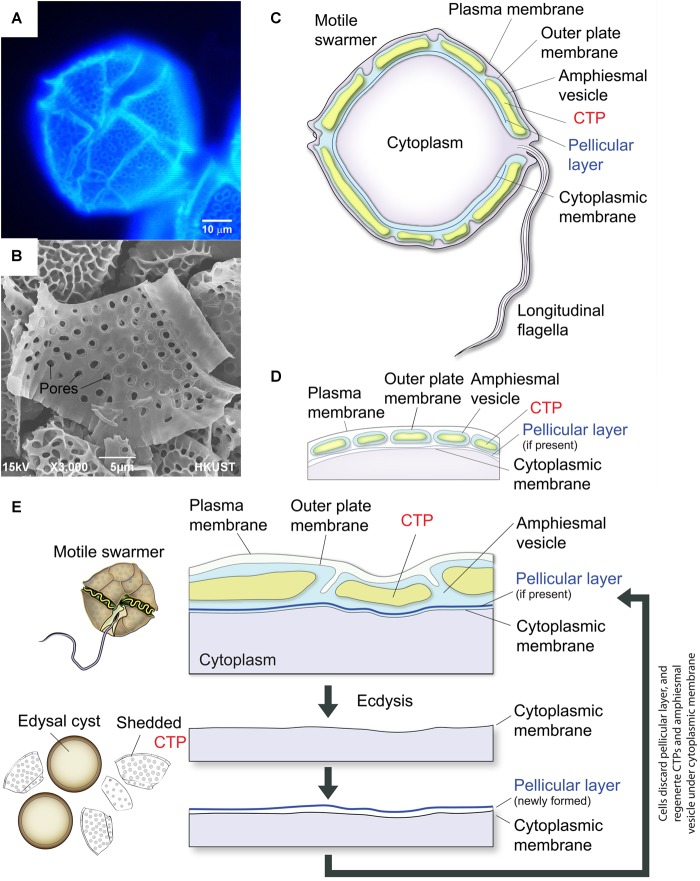
Diagrammatic representation of thecate dinoflagellate cell wall during ecdysis. **(A)** Fluorescence photomicrograph of CFW-stained CTPs in a *Lingulodinium polyedrum* cell. **(B)** Scanning electron microscopic image of isolated CTPs from *L. polyedrum*. Front and back of CTPs had different three-dimensional structures. **(C)** Schematic diagram of the intracellular cell wall of a typical thecate dinoflagellate based on Morrill and Loeblich (1983). The cell coverings (amphiesma) of thecate dinoflagellates consist of CTPs, pellicular layer (if present) and multiple membranous layers. The plasma membrane or plasmalemma defines the borders of the cell (the outermost layer). “Cytoplasmic membrane” here means the membrane in immediate contact with the cytoplasm (the innermost membrane). CTPs and pellicle (if present) are enclosed within a single-membrane-bound AV lying beneath the plasma membrane. **(D)** There are considerable discrepancies in the interpretation of amphiesmal (cell wall) arrangement, which is likely different for different species. The amphiesmal arrangement first described by Dodge and Crawford (1970) and Loeblich (1970) suggested that individual CTP are housed within separate, individual AVs. Amphiesmal arrangement likely changes with cell-cycle and life-cycle dynamics. **(E)** CTPs are shed during ecdysal cyst formation, with rapid formation of pellicular layer (cyst wall) outside the cytoplasmic membrane. Soon after the cells escaped from the pellicles, they regain motility and regenerate CTPs in the newly formed AV.

Dinoflagellates have profound ecological importance, with many species being significant members of phytoplankton, as both primary producers and grazers, as well as causing regular seasonal blooms and red tide. Symbiotic dinoflagellates of corals form the primary productivity base in coral reef ecosystems (Davy et al., 2012; Hart et al., 2015; Hu et al., 2015). Cellulose is commonly deposited on extracellular matrix during cell wall formation. The “internal” cell wall (or amphiesma) in dinoflagellates consists of two cortical intracellular layers: (i) the highly patterned CTPs (Figure 1B–D) in AV (thecal vesicle or alveoli) (Morrill and Loeblich, 1983; Bogus et al., 2014) in thecate species and (ii) the pellicular layer with no or questionable cellulose content (Morrill and Loeblich, 1981). CTPs, the prominent cortical structures in thecate dinoflagellates, have precise architecture and dimensions, which are used for taxonomic differentiation between species. CTPs are commonly regarded to have protective functions and our nanoindentation study suggested they have similar mechanical properties to soft wood (Lau et al., 2007). CTPs can be up to microns in thickness and 30–50 microns in width (Morrill and Loeblich, 1983), and representing substantial weight of the dinoflagellate cell and renewable carbon. Electron photomicrographs of freeze-fracture replica of dinoflagellate cellulose-synthesizing layers revealed a new linear type of CSCs, which were irregularly spaced and form two rows, that has not been found in other organisms (Sekida et al., 2004).

Alveoli, the signature cortical membrane-bound sac(s) shared by members of the Alveolates (Cavalier-Smith, 1993), usually subtend the plasma membrane and are either having no CTPs (in apicomplexans, ciliates, and athecate dinoflagellates) or filled with CTPs in thecate dinoflagellates (Figure 1C,D). Alveoli, which store Ca^2+^ for rapid release (Stelly et al., 1991), serve as scaffold for the machinery driving cell locomotion (gliding or cilia beating) and host cell invasion in both apicomplexans and ciliates (Stelly et al., 1991; Volkmann et al., 2012). However, little is known about the functions of dinoflagellate alveoli and there is very little information as to CTP biogenesis. The subcellular nature of CTPs implicated the involvement of very different biogenesis mechanisms, as compared to plant cell wall. The absence of CesA1 orthologs in other alveolate members also implicated CS in CTP could involve other members of the superfamily.

Life histories of most dinoflagellates are complex with high degree of plasticity and multiple life-cycle stages, composing of different pathways through the alternation of asexual and sexual reproduction (Litaker et al., 2002). The common vegetative stages in the life histories of most dinoflagellates are haploid motile cells (e.g., swarmer cells, mastigote cells), which can differentiate into different asexual and sexual life-cycle stages, including formation of cysts (Pfiester and Anderson, 1988; Bravo et al., 2010; Bravo and Figueroa, 2014). Life-history-stage transitions of dinoflagellates, including the formation of pellicle and sexual cysts and excystment events, are important determinants in the initiation and dynamics of dinoflagellates in coastal ecosystems, including those of red-tides (Anderson et al., 2012); the physiological and environmental conditions for which had received much attention. Transition from the motile “mastigote” stage to non-motile “coccoid” stage is also essential to the establishment of coral-zooxanthallae symbiosis (Fitt and Trench, 1983). “Coral bleaching” involves the loss of this non-motile coccoid stage. The common element in many of these dinoflagellate life-history-stage transitions is reversible differentiation between motile and immotile cells, involving regeneration of the complex cell coverings (termed amphiesma) and deflagellation (Höhfeld and Melkonian, 1992); hence very often referred to “cyst” formation.

In response to certain environmental conditions (e.g., turbulences, photoperiod), and including in response to adverse environmental conditions (e.g., temperature and mechanical stresses), many dinoflagellates will form immotile cells termed ecdysal, temporary, or pellicle cysts (Adamich and Sweeney, 1976; Sweeney, 1976; Bricheux et al., 1992). Ecdysal cysts can also be involved in sexual (sexual temporary/ecdysal cysts) reproduction (Figueroa et al., 2006; Bravo and Figueroa, 2014). During the process of encystment, many morphological changes including shedding of their cell coverings occur (Morrill, 1984), and pellicle cysts are very often also referred to as “ecdysal” cysts (Figure 1E). On return to favorable environmental conditions, the swarmer cells-pellicle cyst transition is readily reversible. Motile-immotile cyst formation, as exemplified by mechanically-induced pellicle cyst formation, involved calcium signaling pathways and could be induced with the circadian hormone melatonin (Balzer and Hardeland, 1991, 1992; Tsim et al., 1997, 1998; Yeung et al., 2006).

*Lingulodinium polyedrum* is the most well-characterized dinoflagellate in terms of its luminescent response to flow (Anderson et al., 1988; Latz and Rohr, 1999; Von Dassow and Latz, 2002). The availability of cyst-generation method (Adamich and Sweeney, 1976; Sweeney, 1976; Bricheux et al., 1992), in combination with CFW-assisted flow cytometry of cellulose content in dinoflagellate cells (Kwok and Wong, 2003), facilitate investigations of CS dynamics and CTP biogenesis during T_c–s_ in *L. polyedrum* in the present study.

Dinoflagellates nuclear genome is extremely large, with many genes present in multiple copies (Shoguchi et al., 2013), implying that antisense-based approach would be more applicable than possible gene knockout method. As strict stoichiometry was enforced in multimeric CesAs within CSCs (Carroll and Specht, 2011; Carroll et al., 2012; Hill et al., 2014), we adopted a gene-knockdown approach in the functional study of dinoflagellate *CesA1*. Our results demonstrated successful gene-knockdown in dinoflagellates and suggested CesA1p-mediated intracellular CS is essential for CTP formation and efficient life-cycle transitions.

## Materials and Methods

### Cell Culture, Ecdysal Cysts Induction, and Regeneration of Motile Swarmers

Dinoflagellate *Karenia brevis* CCMP 2229, *L. polyedrum* CCMP 1931 and *Karlodinium micrum* CCMP 1975 were obtained from the Provasoli-Guillard National Center for Culture of Marine Phytoplankton (CCMP) and cultured at 18°C with L medium as recommended by CCMP.

Formation of immotile ecdysal cysts, also termed pellicle cysts or temporary cysts, involves shedding of old cell wall including CTPs, followed by regeneration of motile swarmer with full complement of amphiesma (Marasovic, 1989; Roy et al., 2014) (Figure 1E). Regeneration of motile swarmers cells from immotile cysts can be readily observed. CTPs regeneration can be accomplished within a relatively short time without complications of old cell wall. We adapted published protocols using mechanical stress (centrifugation) for stimulating ecdysal cysts induction (Adamich and Sweeney, 1976; Sweeney, 1976; Bricheux et al., 1992), and harvested the cells strictly at the same circadian time (2 h before dark phase) to increase experimental repeatability. To induce ecdysal cyst formation, *L. polyedrum* cells were gently harvested by centrifugation (700 × *g* for 5 min) and re-suspended carefully in fresh medium (T = −2). Cells were then incubated at 18°C with illumination.

### Cloning of *KbCesA1* and *in silico* Analyses

Expressed sequence tags (ESTs) encoding partial dinoflagellate CesA sequences were identified from our in-house transcriptomes and NCBI (National Center for Biotechnology Information) EST database. Matches which showed significant homology to oomycetes cellulose synthase 3 (with e-value smaller than 9e^−10^ in the EST database (Accession Nos. CO062648.1, CO063591.1, EX968983.1, EX968982.1, EX870214.1, EX870215.1, FK839351.1, FK841069.1, FK839351.1, and FK839422.1) were extracted for initial contig assembly (*Kb*CesA1), which was used as template to confirm overall homology between dinoflagellate orthologs. *LpCesA1*, CesA1 ortholog from *L. polyedrum*, was obtained from GenBank (GABP01065332.1, e-value = 0.0) by a BLAST search (tblastn) against transcriptome shotgun assembly (TSA) database using *Kb*CesA1 amino acid sequences as query.

For RT-PCR (reverse transcription-PCR), total RNA and cDNAs were prepared from *K. brevis* as previously described (Kwok and Wong, 2010). 5′ and 3′ UTR (untranslated region) of *Kb*CesA1 was amplified by using transcript-specific primers, CSL primer and oligo-dT primer (Supplementary Figure S1). Gene-specific primers were designed from assembled contigs and used to amplify the full-length *KbCesA1* gene from *K. brevis*. All the primers used were listed in Supplementary Table S1.

Conserved domains in the CesA orthologs were analyzed using the NCBI Conserved Domain search program and Conserved Domain Architecture Retrieval Tool at NCBI. Putative transmembrane domains were predicted using TMHMM Server Version 2.0.

### Gene-Knockdown Study

CesA1 antisense-oligonucleotide (ODN) (5′-TTCCACAGTCCGTTCTCG-3′) were designed using principles of nucleic acid thermostability by selecting 18–20-bp antisense fragments (Liao et al., 2013) from the *L. polyedrum* CesA (*LpCesA1*) sequences (corresponds to position 1185 to 1202 bp in *LpCesA1* cDNA (2577 bp in total) obtained from NCBI GenBank (GABP01065332.1). Control ODN was the scrambled sequence (5′-GTTAGCATAGAACCTACA-3′). We adapted a spheroplast (ecdysal cysts)-based transfection method (Kwok et al., 2007) with Lipofectamine^®^Reagent 2000 (Invitrogen) for the antisense ODN-mediated knockdown of LpCesA1p. Ecdysal cyst were generated as described above. For each transfection sample, oligomer-Lipofectamine 2000 complexes were prepared by mixing 300 μl L medium, 300 μl 12 nmole antisense or scrambled ODN and 30 μl Lipofectamine Reagent 2000 (Invitrogen). The mixture was incubated for 30 min at room temperature (with rotation), before incubating with 300 μl spheroplasts (original culture: 800 ml, final cell number: ∼1.8 × 10^6^) for another 1 h at room temperature (with gentle rotation). The transfected cells were washed twice (1,200 × *g* for 3 min at room temperature) with L medium and resuspended in fresh L medium at a density of 1.25 × 10^4^ cells ml^−1^ before incubation in light at 18°C. Cells were accordingly harvested at 12, 20, and 24 h post-transfection. To monitor the uptake of antisense ODN, ecdysal cysts were also transfected with fluorescein (FITC; 5′-end)-labeled CesA1 antisense-ODN (Invitrogen) in pilot test (Supplementary Figure S2).

### Flow Cytometric Analysis

All flow cytometric analyses were performed based on previous protocols with cells stained with 0.1% (w/v) CFW (Kwok and Wong, 2003). All measurements were performed on a BD FACSAria^TM^ IIIu cell sorter (BD Biosciences). All flow cytometric data were analyzed using the software WinMDI (version 2.8; The Scripps Research Institute) running “total” events (10,000). To estimate the percentage of cells and CFW fluorescence intensity of specific population in a sample, the specific regions corresponding to different CFW peaks were gated on dot-plot flow cytograms using the “Region” function available in the WinMDI software.

### Updegraff Cellulose Assay

In Updegraff assay, cell pellet (>1 × 10^4^ cells per sample) were heated at 100°C for 30 min in 1 ml acetic/nitric acid reagent (concentrated acetic acid:concentrated nitric acid:water = 8:1:2). This would have degraded polysaccharides in the sample, including callose and non-crystalline cellulose (Updegraff, 1969; Bulone, 2007), and will only estimate the amount of crystalline cellulose. After washing twice with 1 ml distilled water, the insoluble pellet was incubated with 1 ml 67% sulfuric acid for 1 h. The amount of glucose (released from breakdown of crystalline cellulose) was measured by a colorimetric method using anthrone reagent (Spiro, 1966; Kumar and Turner, 2015). One-tenth (100 μl) of the sulfuric acid-dissolved sample was mixed with 900 μl of anthrone reagent, before boiling for 10 min and absorbance measurement at 630 nm. A standard curve was constructed by using different amount of cellulose powder (Avicel PH-101, Sigma-Aldrich).

### Quantitative Real-Time PCR (qPCR) Analysis

Total RNA was isolated using Trizol reagent (Invitrogen) and the first-strand cDNA was synthesized by SuperScript^TM^ II reverse transcriptase (Invitrogen life technologies) with oligo dt adapter primer (5′-GGCCACGCGTCGACTAGTACTTTTTTTTTTTTTTTTT-3′) according to the manufacturer’s instructions (Kwok and Wong, 2010). qPCR conducted in triplicates in a 10 μl reaction volume containing 1 ng of first-strand cDNA, 100 nM gene specific primers and 1X QuantiFast SYBR Green PCR Master Mix (Qiagen). The Applied Biosystems 7500 Fast Real-Time PCR System was used to quantify expression of the genes. The thermal cycling condition was shown as followed: 50°C for 2 min, 95°C for 10 min, 40 cycles for 95°C for 10 s and 56°C for 30 s. Glyceraldehyde 3-phosphate dehydrogenase (GAPDH) gene from *L. polyedrum* (AF028562.1) was used as endogenous control as its expression was the most stable among several selected reference genes (Supplementary Figure S3). The GAPDH gene has been reported as the most stable endogenous control in dinoflagellates (Guo and Ki, 2012; Shi et al., 2013), and it has been widely used as reference gene in dinoflagellates (Luo et al., 2017; Zhang et al., 2017). Relative expression levels were determined by the 2^−ΔΔCt^ method (Livak and Schmittgen, 2001). Gene expression values were normalized with the cycle threshold geometric mean values of the GAPDH reference gene (Teste et al., 2009). All the primers used in qPCR are presented in Supplementary Table S1.

### Phylogenetic Analysis of Cellulose Synthase Orthologs

Predicted amino acid sequences of dinoflagellate CesAs and other CesA orthologs from GenBank database were used for phylogenetic analyses. Multiple protein sequence alignments were performed on both full length regions and conserved substrate binding domains (U1 to U4; the cytoplasmic domain between the second and third TMD) by ClustalW in the MEGA7 software package (Pairwise and multiple alignment parameters – Gap opening penalty 10, Gap extension penalty 0.1, delay divergent sequences 30% and no use of a negative matrix) (Tamura et al., 2011). Alignments were further adjusted by eye to minimize the effects of insertion/deletion events on the analysis. Phylogenetic trees were constructed based on the alignments by the maximum-likelihood method using Tamura-Nei model in MEGA7 (Test of phylogeny options: Bootstrap 1000 replicates; Rates among sites: Uniform rates; Gaps/Missing Data Treatment: Complete Defleletion). The bootstrap values (percentage) for each branch point were shown in italics.

### Immunoblotting and Antibody Preparation

For immunoblot analyses, whole-cell lysates were prepared as described previously (Kwok and Wong, 2010). Protein loading and relative expression levels was verified by probing the same blot (region around 55 kDa) with anti-alpha-tubulin mouse monoclonal (1:3000 dilution) (Sigma-Aldrich) and HRP-conjugated anti-mouse IgG (1:5000 dilution). Labeled protein bands were detected with the Clarity Western ECL Substrate (BIO-RAD) according to the manufacturer’s manual. Band intensities were determined using ImageJ software (NIH) (Sheffield, 2008). CesAp expression levels were normalized to alpha-tubulin level, only bands within the same gel were compared.

Recombinant polypeptides composing the N-terminal region of *Kb*CesA1p (amino acids 1 to 107), produced in *E. coli* (with His-tag), and purified by Ni-NTA resin (under denaturing conditions, QIAexpressionist; Qiagen), was used as immunogens for the generation of anti-CesA1p (anti-KbCesA1p) polyclonal antibody. Rabbit polyclonal antibodies were generated in the Animal and Plant Care Facility at the Hong Kong University of Science and Technology following institutional and National Institutes of Health guidelines. Immunization was carried out following published protocols (Harlow and Lane, 1988). All immunological studies employed antigen affinity-purified antibodies (Robinson et al., 1988).

### Calcofluor White Staining, Cryosectioning, Immunostaining, and Confocal Microscopy

Calcofluor white staining, which stained both non-crystalline and crystalline cellulose, is a method commonly used for estimating cellulose abundance, including dinoflagellate cells which had no callose (Kwok and Wong, 2003; Fujise et al., 2014). Ecdysal cysts were first fixed in 2% (w/v) paraformaldehyde overnight at 4°C before replacing with PBS containing 10 μg ml^−1^ CFW. Photomicrographs were taken from a Leica fluorescent microscope (DMLS) equipped with a digital camera (INFINITY 3, Lumenera).

In order to visualize the localization of LpCesA1p within the internal cell wall, cryosections (5 μm) were prepared as described previously (Soyer-Gobillard et al., 2002), except that infiltration of cell pellets with polyvinylpyrrolidone (PVP)/sucrose (20% (w/v) PVP, 1.7 M sucrose) was performed by a stepwise manner (Ausseil et al., 1999). Confocal imaging employed a Leica TCS SP5 II confocal system. Whole cell immuno-labeling was performed as described previously (Soyer-Gobillard et al., 2002; Kwok and Wong, 2010).

### Chemical Inhibitors

The herbicide DCB is a widely-used chemical inhibitor for studying the effects of CS inhibition in different cellulose-synthesizing organisms (Arad et al., 1994; Delmer and Amor, 1995) and our previous study verified its action on CS inhibition in dinoflagellates (Kwok and Wong, 2003). Stock solutions of DCB (Sigma-Aldrich) was prepared in dimethyl sulfoxide (DMSO), with a final DMSO concentration of 0.0625% (v/v) and DCB concentrations at 100 μM. All chemicals were from Sigma-Aldrich unless otherwise stated.

### Statistical Analyses

All experiments were conducted at least in triplicates. An unpaired *t*-test or one-way ANOVA analyses of variance with Bonferroni/Newman–Keuls post-tests were performed for statistical tests using GraphPad Prism. Results were considered significant when *P* < 0.05.

### Accession Numbers

The full-length sequence of *KbCesA1* has been submitted to the GenBank database under accession number KY352307. EST sequences used for assembling the *KbCesA1* contig (CO062648.1, CO063591.1, EX968983.1, EX968982.1, EX870214.1, EX870215.1, FK839351.1, FK841069.1, FK839351.1, and FK839422.1). Other ORFs used for phylogenetic tree construction are*: L. polyedrum* (*LpCesA1* [GABP01065332.1]), CesAs from *Agrobacterium tumefaciens* CCNWGS0286 (*At*BcsA [EHH05269.1]), *Albugo laibachii* Nc14 (*Al*CesA3 [CCA19356.1]), *Anabaena variabilis* ATCC 29413 (*Av*CcsA [ABA21191.1]), *Arabidopsis thaliana* (*At*CesA [AAC29067.1]), *Burkholderia vietnamiensis* G4 (*Bv*BcsA [YP_001119200.1]), *Ciona intestinalis* (*Ci*CesA [NP_001041448.1]), *Crocosphaera watsonii* (*Cw*CcsA [EHJ15054.1]), *Cyanothece* ap. PCC 7424 (*C*CcsA [YP_002376652.1]), *Eucalyptus grandis* (*Eg*CesA [ABY25278.1]), *Gluconacetobacter sucrofermentans* (*Gs*BcsA [BAA31463.1]), *Gossypium hirsutum* (*Gh*CesA [AAB37767.1]), *Griffithsia monilis* (*Gm*CesA [ADK77974.1]), *Mesotaenium caldariorum* (*Mc*CesA [AAT48369.1]), cyanobacteria *Nostoc punctiforme* (*Np*CcsA [WP_012408188.1]), *Oikopleura longicauda* (*Ol*CesA [BAJ65323.1]), *Oikopleura dioica* (*Od*CesA [CAJ43274.1]), *Oryza sativa* Japonica Group (*Os*CesA [AAO41140.1]), *Phytophthora sojae* (*Ps*CesA3 [ABP96908.1]), *Phytophthora infestans* (*Pi*CesA3 [ABP96904.1]), *Populus tremula* × *Populus tremuloides* (*Pt*CesA [AAT09896.1]), *Pyropia yezoensis* (*Py*CesA [ABX71734.1]), *Pseudoperonospora cubensis* (*Pc*CesA3 [AEC45570.1]), *Rhizobium leguminosarum* bv. *Saprolegnia monoica* (*Sm*CesA3 [ACX56231.1]), *Synechococcus elongatus* PCC 7942 (*Se*CcsA [YP_401168.1]), *Synechococcus* sp. PCC 7002 (*S*CcsA [ACB00100.1]), *Thermosynechococcus vulcanus* RKN (*Tv*CcsA [BAJ61014.1]), *Gloeomargarita lithophora* (*Gl*CcsA [APB34729.1]).

## Results

### *In silico* Analysis of Dinoflagellate Cellulose Synthase Orthologs

**FIGURE 2:**
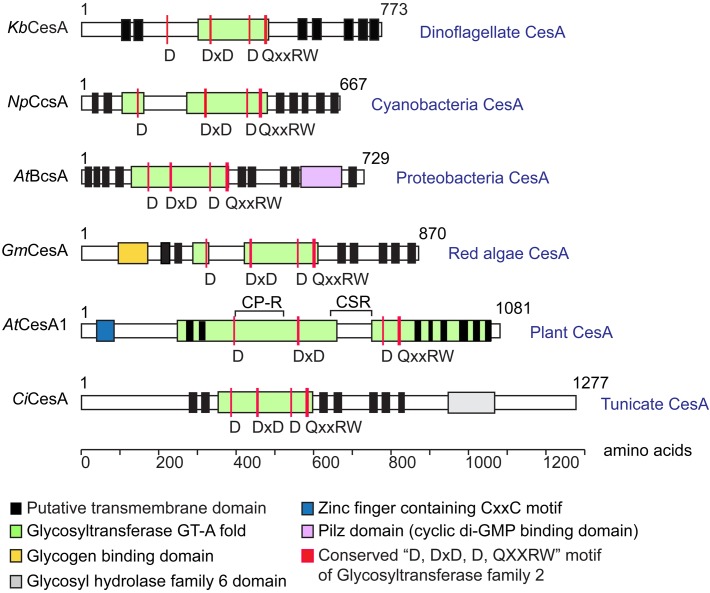
Predicted domains arrangement of selected CesA orthologs. Domain organizations of CesAs of *Karenia brevis* (*Kb*CesA, KY352307), *Nostoc punctiforme* (*Np*CcsA, YP_001865112.1), *Agrobacterium tumefaciens* (*At*BcsA, NP_357298.1), *Griffithsia monilis* (*Gm*CesA, ADK77974.1), *Arabidopsis thaliana* (*At*CesA1, AEE86053.1), and *Ciona intestinalis* (*Ci*CesA, NP_001041448.1) are given for comparison. Dinoflagellate *Kb*CesA contained the conserved CesA catalytic motif (D, DxD, D, and QxxRW), amid low levels of overall sequence similarity with other CesA orthologs at the catalytic domain (e.g., ∼20–25% with CesAps from *Arabidopsis*; 83% between *Kb*CesAp and the *LpCesA1*p). Dinoflagellate CesAs encode predicted polypeptides (∼90 kDa) similar in size to bacterial CesA (BcsA), but much smaller than other eukaryotic CesAps.

Dinoflagellate CesAs encoded predicted polypeptides (∼90 kDa) similar in size to bacterial CesAps (BcsA), but much smaller than plant CesAps (Figure 2). Dinoflagellate CesA orthologs contained the conserved glycosyltransferase catalytic motif (D, DxD, D, and QxxRW) (Saxena et al., 2001; Saxena and Brown, 2005) (Figure 2), and showed intraspecies similarity (in percentage identity) at ∼83% and interspecific similarity at ∼20–25% (with plants). Dinoflagellate CesAs contained at least seven predicted transmembrane domains (TMDs), with two or more TMDs prior to and five TMDs distal to the predicted intervening glycosyltransferase domain (Figure 2); an organization pattern observed in many linear-type CesAs. Instead of having long PilZ-domain (c-di-GMP regulation) in bacterial CesAps (BcsA) (Morgan et al., 2014), the predicted dinoflagellate orthologs had no recognizable motifs or domains in the conserved c-terminal distal to the last predicted TMDs (Figure 2).

By rooting the CesAs tree with the cyanobacterial CesAs (CcsA2 clade) and adding the sequences from the closest plastid ancestor [*Gleomargarita*; a freshwater cyanobacteria (De Vries and Archibald, 2017; Moreira et al., 2017; Ponce-Toledo et al., 2017; Sánchez-Baracaldo et al., 2017)], two phylogenetic trees with similar topology were resulted from selected CesA orthologs (Supplementary Figures S4A,B). Consistent with previously described, two distinct cyanobacterial CesA lineages (CcsA1 an d CcsA2) form sister clades to known CesAs of eukaryotes (plants, green and red algae, oomycetes, and dinoflagallates) and prokaryotes (proteobacteria), respectively (Supplementary Figures S4A,B) (Nobles and Brown, 2004). Within the eukaryotic clade, the dinoflagellate lineage pre-dated other putative linear-type CesA clades of red algal and oomycete (Stramenopiles) orthologs, and shared a node which lineaged CesA orthologs of plants and green algae (Supplementary Figures S4A,B). It was postulated that a common ancestor of Alveolata (including dinoflagellates), Stramenopiles (oomycetes) and Haptophyta acquired their cellulose-synthesizing machinery through a secondary endosymbiosis event involving a red alga (Popper et al., 2011). However, there were no cellulose in other Alveolates (ciliates and apicomplexans), and our search for apicomplexan and ciliate CesA orthologs did not result in any positive results, implicating CesA orthologs in other alveolates were either lost or dinoflagellate acquired their CesAs from another source. Further analysis of dinoflagellate CesA1 origin and diversification would be interesting with significantly more samples.

### Immunolocalization of CesA1p in Dinoflagellate Cell Wall

**FIGURE 3:**
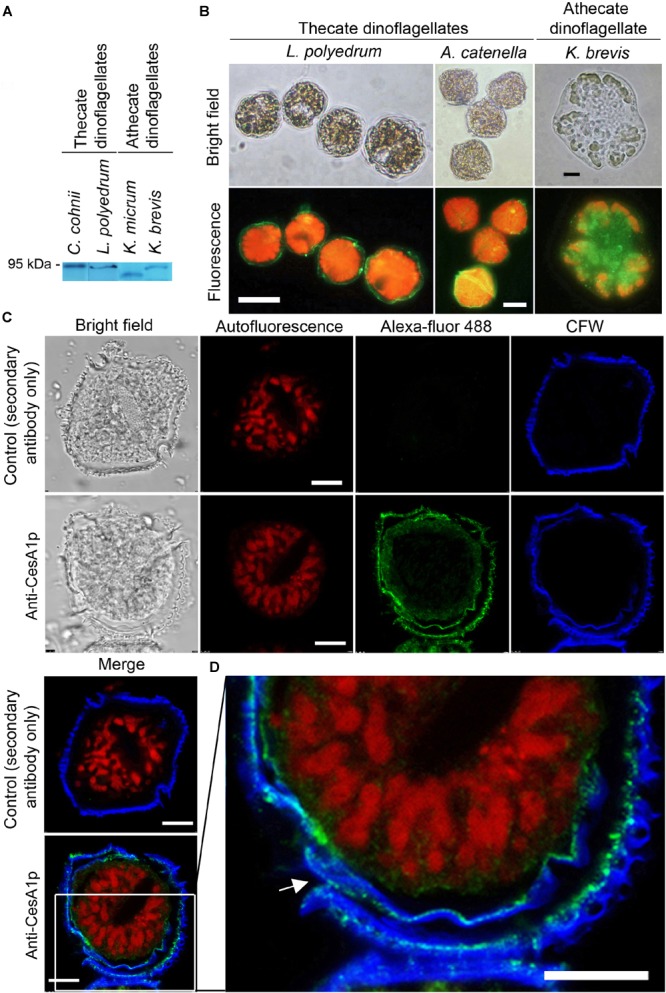
Cortical localization of CesA1p in dinoflagellates. **(A)** Western blot analysis of cell lysates from thecate and athecate dinoflagellates. **(B)** Fluorescence photomicrographs of whole cells labeled with anti-CesA1p antibody. **(C)** Confocal images showing the immunofluorescence-labeled cryosections prepared from *L. polyedrum* cells. Green fluorescence (Alexa Fluor 488 goat anti-rabbit IgG), corresponding to CesA1p, was localized to margins of overlapping CTPs; the continuity over one CTP was demarcated in enlarge image in **(D)**. CFW fluorescence (Blue) also localized to this layer. CTPs are not flattened but three-dimensional structures with different front and back side (Figure 1A,B). Red fluorescence corresponded to the auto-fluorescence of chloroplast pigments. **(D)** CesA1p immunofluorescent pattern in cortical region with higher magnification. Its continuity around a CTP was highlighted with arrow. Antigen-purified anti-CesA1p antibody was used throughout the experiments. Scale bars represent 10 μm.

Calcofluor White staining identified the major armor-like CTP layer, which composed of individual CTPs in flattened AVs (Figure 1A). Whole-cell and confocal immunofluorescence imaging of CesA1p using affinity-purified anti-CesA1p antibody (N-terminal targeted), which was immuno-reactive to polypeptide bands of the expected size (∼90 to 95 kDa) in cell lysates from different dinoflagellates (Figure 3A), revealed cortical localizations (Figure 3B,C). Confocal immunofluorescence imaging of CesA1p (in cryosections) exhibited cortical localization with CFW staining in the cortical layers, with CesA1ps location surrounding CTP membranes, hence giving the appearances of two layers (Figure 3C,D). The inner pellicular layer (in regenerating pellicle cyst) apparently had no detectable CFW staining in *L. polyedrum.* However, higher resolution microscopy would be required to unequivocally conclude the absence of cellulose in pellicular layer, which is dynamic in relation to ecdysal stages, and was previously considered to have low or no cellulose.

### Cellulose Synthesis and CesA1 Expression During Cyst-to-Swarmer Transition

**FIGURE 4:**
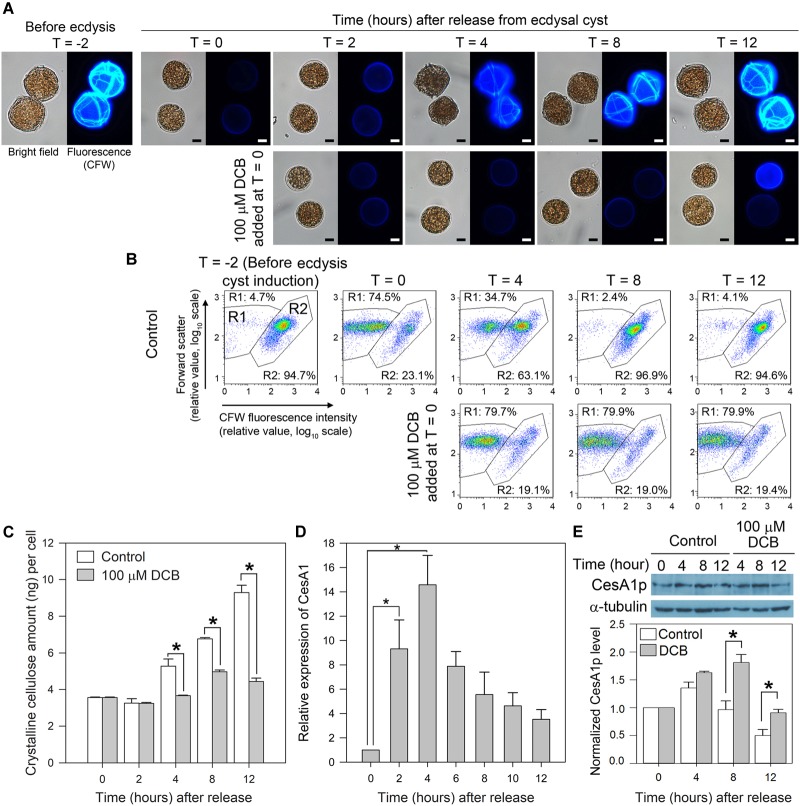
LpCesA1 expression and cellulose synthesis (CS) during cyst-to-swarmer transition. **(A)** Fluorescence photomicrographs of *L. polyedrum* cells (stained with CFW) during cyst-to-swarmer transition (T_C–S_). Control contained 0.0625% (v/v) of DMSO, the vehicle for DCB. Ecdysis resulted in a decrease in cellulose content (CFW fluorescence) from T = –2 to T = 0. **(B)** CFW (cellulose)-stained cells were distributed in R1 and R2 regions in relation to their relative CFW fluorescence intensity and cell size (forward scatter). R1, with less CFW staining should be the ecdysal cyst whereas the R2 should be the swarmer cells (and cyst with attached cell wall). However, these two populations were complicated with some shed cell-wall attaching to the ecdysal cyst. DCB served as a negative control for cells which did not increase in cellulose content, verifying sub-population of R2 cells had detached cell wall (some R2 cells had larger FSc). Almost all cells had higher level of CFW-staining by T = 8–12, suggesting continuous cell wall growth after regaining motility (between T = 4 and T = 8 in different cells). The 74.5% (R1) was thus an underestimate for ecdysal cyst, as no swarmer cell could be identified in DCB-treated cells; transfection involved lipofectamine and additional centrifugation, nor did visual observation reveal any motile cells in T = 0 sample. **(C)** Crystalline cellulose levels per cell during T_C–S_. **(D)** Levels of *LpCesA1* transcripts during T_C–S_. **(E)** Anti-CesA1p immunoblot of cell lysates collected during T_C–S_. Control experiment: DCB (100 μM) completely inhibited the reformation of CTPs **(A)** and significantly (*P* < 0.05) decreased cellular cellulose content **(C)** throughout the experiment. The residual cellulose likely corresponded to attached old cell wall which were not removed from vegetative cells after washings. Scale bars represent 10 μm. Relative expression pattern reports normalized signals of CesA1p with alpha-tubulin signals. Data represent means ± SE of triplicate experiments. Asterisks (^∗^) indicates significantly different from control (*P* < 0.05).

Two hours after centrifugation (T = 0), most cells retracted from their old cell wall layers and formed immotile ecdysal cysts (Figure 4A). Flow cytometric estimation of cellulose content (CFW fluorescence) dropped with shedding of old amphiesmal layer during ecdysis, which recovered upon regeneration of swarmer cells within 8–12 h. The overall microscopic observations and interpretation corresponded with CFW-flow cytograms (Figure 4A,B). In flow-cytometic dot plots (Figure 4B), CFW (cellulose)-stained cells were distributed in R1 and R2 regions in relation to their relative CFW fluorescence intensity and cell size (forward scatter). Before ecdysis, the majority (∼95%) of the swarmer cells (with heavily stained CTPs, Figure 4A) were distributed in the R2 region (high CFW fluorescence) in flow-cytometric dot plots (Figure 4B). R1 with less CFW staining was resulted to be the major population, following induced ecdysis (centrifugation). However, these two populations were complicated with some shed cell-wall attaching to the ecdysal cyst. The 74.5% (R1) was thus an underestimate for ecdysal cyst, as no swarmer cell could be identified in DCB-treated cells. DCB-treated cells remained spherical, non-motile (as pellicle cysts) with no CTPs (nor swarmer cells) throughout the experiment (Figure 4A), as reported previously (Kwok and Wong, 2003). DCB treatment served as a negative control to demonstrate sub-population of R2 cells have attached shed cell wall, as R2 persisted with DCB treatment despite no swarmer cells.

*LpCesA1* transcript was upregulated by ∼9 times at T = 2 and peaked at T = 4 (increased by 14 times when compared to T = 0) at early stage of T_C–S_, and remained high throughout the CTPs regeneration process (Figure 4D). Relative low levels of LpCesA1p and cellulose content were observed between T = 2–4 h (Figure 4C,E and Supplementary Figure S5). Relative LpCesA1p level increased 40% by 4 h and peaked at T = 4 (Figure 4E), preceding the increase in cellular cellulose content (T = 12 h) (Figure 4C). At T = 12 h, the R2 population returned back to > 90% (Figure 4B). Most of the cells regained their demarcated CTPs (with strong CFW signals and clear CTP margin) (Figure 4A) and restored their motility, indicating successful regeneration of flagella and cell wall. This orchestrated schedule of *LpCesA1* transcription, LpCesA1p expression, CS and CTP formation would be highly suitable for spatial-temporal study of linear-type CS, as well as provisioning a window (T = 2 to 4 h) for possibly enrichment of active CSCs.

### Knockdown of CesA1p Caused Severe Defects in CTPs and Impeded Cyst-to-Swarmer Transition

**FIGURE 5:**
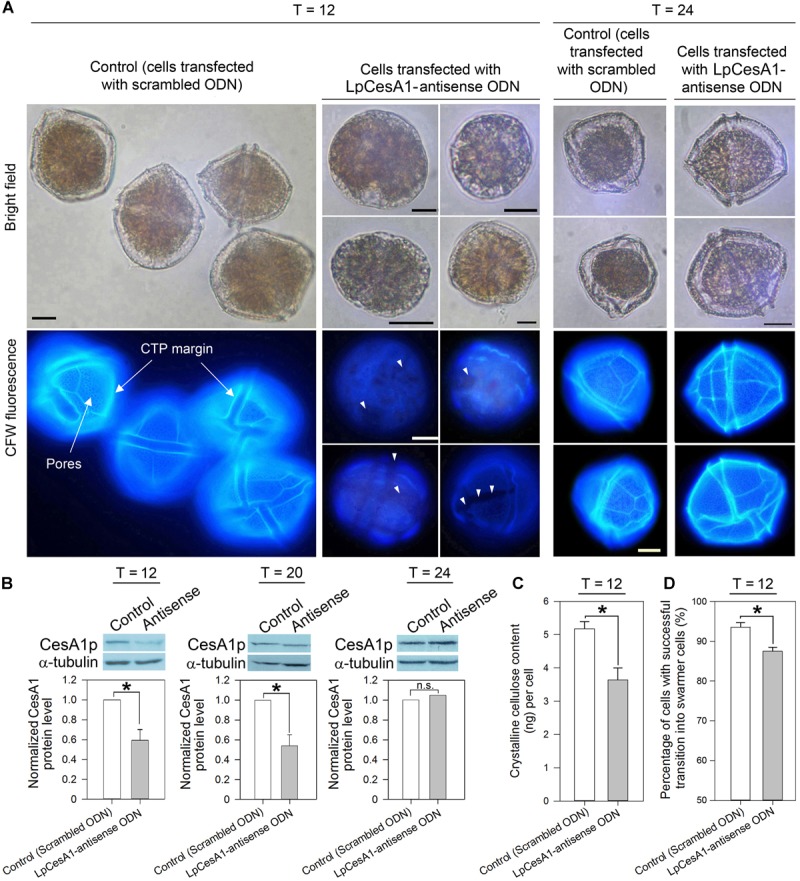
LpCesA1p-knockdown affected CTP biogenesis and cyst-to-swarmer transition. **(A)** Fluorescence photomicrographs of CFW-stained cells transfected with antisense ODN and scrambled ODN (mock-transfected group) collected at 12, 20, or 24 h after released from ecdysal cysts. At T = 12, cells with severely malformed CTPs (with no clear margin, no pores and weaker CFW staining) were observed in the antisense-ODN treatment. Scale bars represent 10 μm. White triangles indicate gaps and cracks on CTP. **(B)** At T = 12, antisense-ODN treatment resulted in significant decrease in the relative LpCesA1p level and **(C)** Crystalline cellulose content (Updegraff assay). **(D)** Antisense-ODN treatment resulted in significant postponement in swarmer cells regenerated from ecdysal cysts, compared with mock-transfected treatment. Asterisks (^∗^) indicates significantly different from control (*P* < 0.05). n.s., not significant. Data represent means ± SE of three replicate experiments.

Cells collected at early T_C–S_ (T = 0 h) were transfected with *LpCesA1*-ODNs, utilizing a liposome-mediated oligonucleotide method (Kwok et al., 2007). At T = 12 h, a 41 ± 10.8% drop in LpCesA1p level corresponded with a significant reduction in cellulose content (30 ± 9.7%), when compared to the mock transfected control (Figure 5B,C). Significantly, this LpCesA1p knockdown caused severe morphological phenotypes (cell number did not decrease significantly), including some spherical cells with no CTPs [with similarity to DCB-treated cells (Figure 4A)], partially or poorly-formed CTPs (with gaps and cracks; Figure 5A, middle panel) and significant T_C–S_ impediment (at T = 12 h, Figure 5D). These variable CTP phenotypes, contrasted with the normal polyhedral-shape in mock transfected control, were likely resulted from different degrees of CesAp knockdown. As LpCesA1p level returned to the original level between T = 20 to T = 24 h (Figure 5B), normal CTP resumed (Figure 5A, right panel) with motile cells in the knockdown treatment. Cumulatively, the severely deformed CTPs in CesA1p knockdown and DCB treatment (Figure 4A) verified the requirement of *CesA1* for CS and CTP biogenesis. It was also likely that some minimal CS was sufficient for T_C–S_, as many cells with malformed CTPs (but not DCB-treated cells) had mobility.

## Discussion

Dinoflagellate life-cycle transitions affect dynamics of algal blooms and reestablishment of zooxanthellae in bleached corals. Motile-immotile switch is a common process in many dinoflagellate life-cycle transition, involving deflagellation and cell wall remodeling. Both ecdysal cyst formation and cellular growth involved calcium signaling and depletion of cortical calcium stores led to cell-wall remodeling (Tsim et al., 1997, 1998; Lam et al., 2005; Yeung et al., 2006). It is likely attributed to share signaling pathway in immotile division cysts formation, which is an integral G_1_-S transition of *C. cohnii* cell cycle (Bhaud et al., 1991) and also involves deflagellation and cell wall remodeling. It is likely not a coincident that alveoli sac, which store Ca^2+^ in ciliates (Stelly et al., 1991), was also the site of major cellulose deposition. Stepwise increase in cellulose content (Kwok and Wong, 2003) and cell cycle-phased *CesA* transcript (Shi et al., 2017) likely reflect coordination with growth at late G_1_ (Kwok and Wong, 2003); it was coincident with growth-induced cyclic ADP-ribose transient which elicited Ca^2+^ release (Lam et al., 2009). It would be interesting to investigate cross-talks between growth-mediated calcium signaling and cell wall deposition in dinoflagellates.

X-ray diffraction patterns of cellulose in CTPs [reviewed in Morrill and Loeblich (1983)] suggested dinoflagellate cellulose were predominantly amorphous (Morrill and Loeblich, 1983), implicating Updegraff assay likely gave underestimates of the total yield of CS. CTPs have three-dimensional structures with different inward- and outward-facing surfaces (Morrill and Loeblich, 1983); the malformed cell shapes with partial CTP formation (Figure 5A) were indicative that correct architecture of CTPs contributed to mechanical support of the thecate dinoflagellate cell. The tightly fitted armor-like plating of CTPs, with multiple membranous layers, likely form a barrier to free diffusion with the continuous pellicular layer. The regularly-spaced outward-facing pits (Figure 1B), regarded as windows for the trichocysts (Vesk and Lucas, 1986; Westermann et al., 2015), likely seconded as channels for environmental communications, as would be the case in non-thecate alveoli-sac.

Cyst-to-swarmer transition plays prominent roles in the dynamics of many dinoflagellate blooms (Bravo et al., 2010). Major cell-wall remodeling are associated with life cycle transitions (Figueroa et al., 2006; Bravo and Figueroa, 2014), including multiple projections and attachment stalk for benthic stages; with dimensions reaching over millimeter in thickness with mechanical property of wood (Lau et al., 2007), it is likely that CTPs contribute to buoyancy adjustment, as well as forming protective armor as commonly depicted. The dinoflagellate *Ceratocorys* interconverted between long-spined and short-spined forms by changing their CTPs shapes with different flow regimes (Zirbel et al., 2002).

Selective inhibition of gene expression by antisense ODN is widely applied in gene function analyses; not only because pleiotropic effects could be minimized (which represent common problems when creating mutants by genetic transformation) (Dinc et al., 2011), but also because of its feasibility to design several ODN targeting a gene family. In spite of the general applicability, the antisense ODN technology has not yet been truly exploited in dinoflagellates, although it was reported that dinoflagellates were capable of taking up single-stranded ODN (Kwok et al., 2007). In the present study, the inhibitory effect of antisense ODNs on CesA1p was strong (40% decrease of CesA1p level; Figure 5B), despite the lack of chemical modification (e.g., phosphorothioate modification) (Ravichandran et al., 2004).

In the present study, the use of antisense generated CesA1p knockdown established the role of *CesA1* in the recovery of thecal plates in the Tc-s by demonstrating the inhibition of plate formation. During cyst-to-swarmer transition (T_C–S_), peaks of CesA1 transcription, CesA1p expression, CS and CTP formation occurred in the corresponding order; ecdysal cyst-regeneration of *L. polyedrum* thus represents a readily available system for *in vivo* functional study of linear-type CSCs. The use of antisense ODN would significantly increase the toolset for investigating gene function in dinoflagellates.

## Author Contributions

WC performed the experiments. WC and AK analyzed the data. JW applied for funding. All authors conceived the project and wrote the manuscript.

## Conflict of Interest Statement

The authors declare that the research was conducted in the absence of any commercial or financial relationships that could be construed as a potential conflict of interest.

## Supplementary Material

FIGURE S1 Cloning of *KbCesA1* from the dinoflagellate *Karenia brevis*. **(A)** Graphical representation of the *KbCesA1* contig assembled from different EST sequences. GenBank accession numbers are indicated on the left. **(B)** Schematic diagram of the strategy of cloning *KbCesA1* by reverse transcription-PCR. 5′ and 3′ untranslated regions (UTR) of *KbCesA1* were amplified by using different primer sets (5′-UTR: CSL and KbCesA-R1 primer; 3′-UTR: KbCesA-F3 and oligo-dT primer). All the primers used are presented in Supplementary Table S1.

FIGURE S2 Pilot transfection test of FITC-conjugated CesA1-antisense ODN. Flow cytograms of *L. polyedrum* pellicle cysts transfected with FITC-conjugated CesA1-antisense ODN (FP). Mock transfection controls include cells only, cells with lipofectamine and cells with FITC-conjugated CesA1-antisense ODN. Ecdysal cyst cells were harvested at T = 0 (immediately after transfection), T = 6 and T = 12 after transfection. Means of fluorescence intensity (log scale) are indicated within parentheses. The cells transfected with the FITC-conjugated CesA1-antisense ODN showed an increase of the mean FITC fluorescence intensity at T = 0 and T = 6 when compared to mock transfection controls, suggesting the antisense ODN could effectively enter the ecdysal cysts using the liposome-mediated oligonucleotide transfection method.

FIGURE S3 GAPDH was the most stable reference gene for quantitative real-time PCR. Box and whisker plots showing the Ct (cycle threshold) values of all tested reference genes. The horizontal line in the middle of each box indicates the median, while the top and bottom borders of the box mark the 75th and 25th percentiles, respectively. The whiskers above and below the box mark the minimum and maximum. Among several commonly used reference genes, the most abundant gene was GAPDH (cycle threshold (Ct) value: ∼15 to 19) and the least was alpha-tubulin (Ct value: ∼18 to 29). The expression stability of all the three candidate reference genes could also be exhibited by the coefficient of variance (CV) values. The CV values were: GAPDH (1.17%), actin (9.59%), and alpha-tubulin (19.34%).

FIGURE S4 Phylogenetic trees constructed with representative CesA orthologs. Maximum-likelihood consensus trees [rooted with cyanobacterial CesAs (CcsA2 clade)] of **(A)** selected CesA orthologs based on full-length sequences and **(B)** conserved U1 to U4 regions. Both trees have the same topology. Dinoflagellate CesA1 orthologs share a node with other putative linear-type eukaryotic orthologs. Bootstrap values of 1,000 replicates are indicated at the nodes. GenBank accession numbers listed in Methods. Bar represents the phylogenetic distance of 0.2 amino acid substitution per site.

FIGURE S5 Expression of CesA1p during early cyst-to-swarmer transition. Anti-CesA1p immunoblot of cell lysates collected during early cyst-to-swarmer transition (T_C–S_). At T = 2, there was no significant increase in CesA1p signals when compared to the control at T = 0. Western blot signals were determined using ImageJ and normalized to the corresponding alpha-tubulin signals. Antigen-purified anti-CesA1p antibody was used throughout the experiments. Data represent means ± SE of triplicate experiments.

TABLE S1 Primers used for the cloning of *KbCesA1* gene and qPCR.

## Abbreviations

AV: amphiesmal vesicle
CesA: cellulose synthase
CFW: calcofluor white
CSCs: cellulose synthase complexes
CTPs: ecllulosic thecal plates
DCB: 2,6-dichlorobenzonitrile
ODNs: antisense oligonucleotides
T_C–S_: cyst-to-swarmer transition.
