# Supplementary figures and images for: Knockdown of Dinoflagellate Cellulose Synthase CesA1 Resulted in Malformed Intracellular Cellulosic Thecal Plates and Severely Impeded Cyst-to-Swarmer Transition
Source: Front Microbiol. 2019 Mar 19;10:546. doi: 10.3389/fmicb.2019.00546 (PMC6433935; doi:10.3389/fmicb.2019.00546)

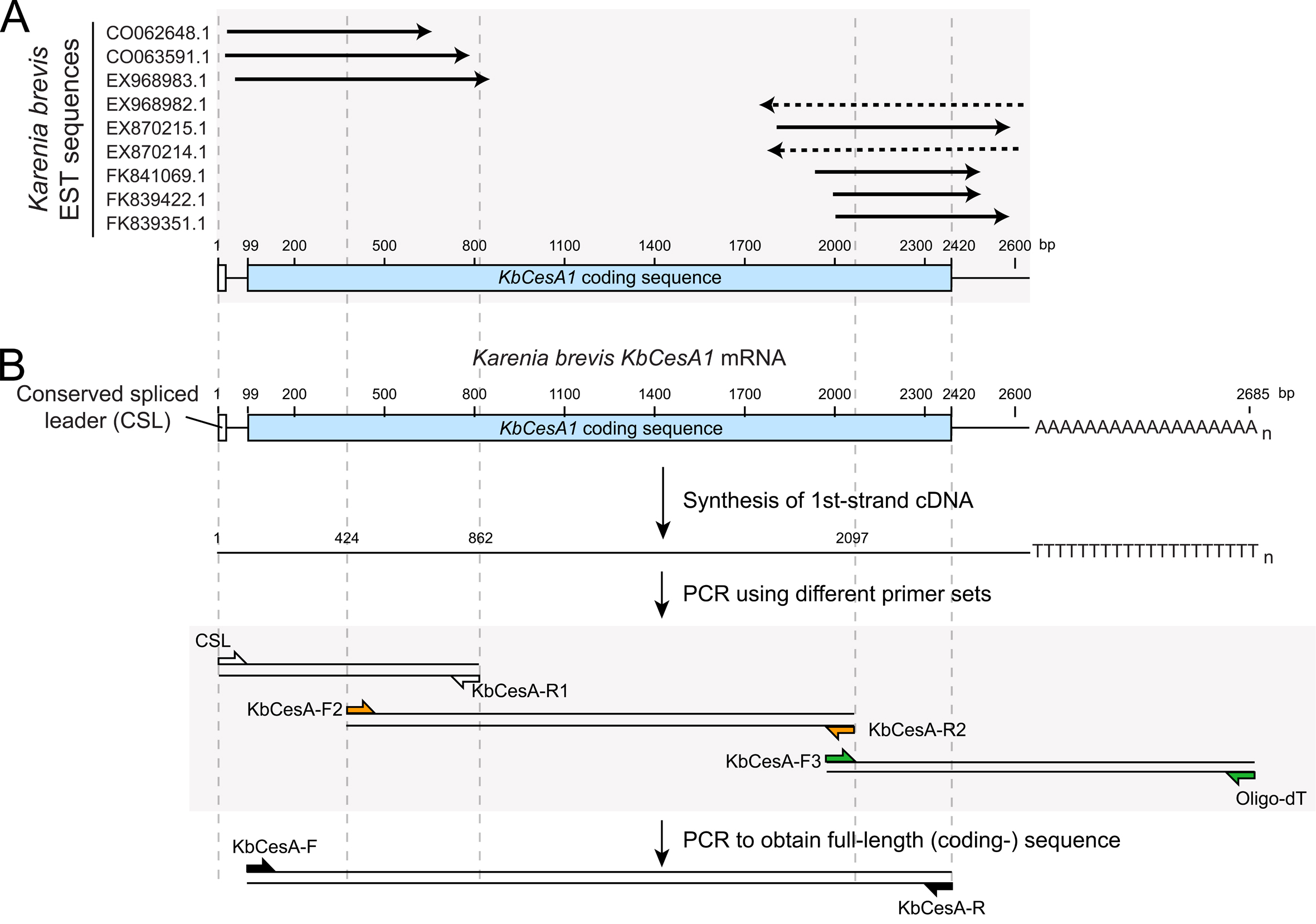

Supplement: FIGURE S1 — Cloning of KbCesA1 from the dinoflagellate Karenia brevis. (A) Graphical representation of the KbCesA1 contig assembled from different EST sequences. GenBank accession numbers are indicated on the left. (B) Schematic diagram of the strategy of cloning KbCesA1 by reverse transcription-PCR. 5′ and 3′ untranslated regions (UTR) of KbCesA1 were amplified by using different primer sets (5′-UTR: CSL and KbCesA-R1 primer; 3′-UTR: KbCesA-F3 and oligo-dT primer). All the primers used are presented in Supplementary Table S1. [file Image_1.JPEG]

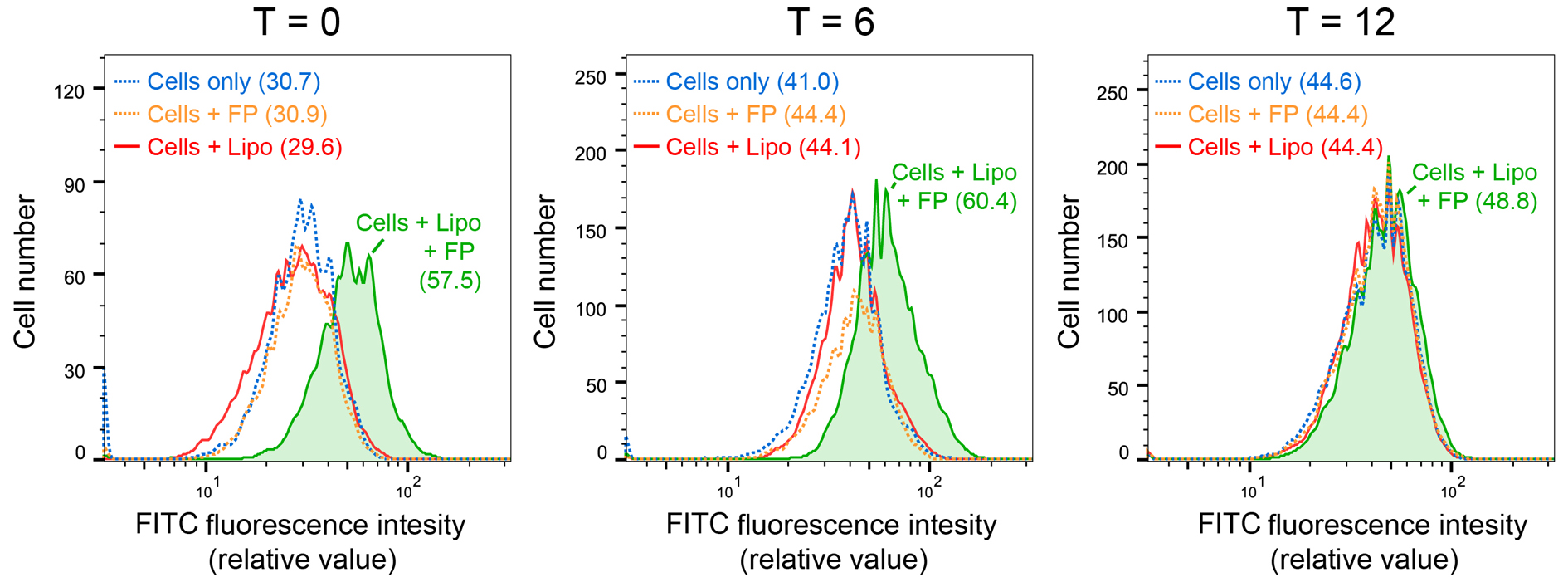

Supplement: FIGURE S2 — Pilot transfection test of FITC-conjugated CesA1-antisense ODN. Flow cytograms of L. polyedrum pellicle cysts transfected with FITC-conjugated CesA1-antisense ODN (FP). Mock transfection controls include cells only, cells with lipofectamine and cells with FITC-conjugated CesA1-antisense ODN. Ecdysal cyst cells were harvested at T = 0 (immediately after transfection), T = 6 and T = 12 after transfection. Means of fluorescence intensity (log scale) are indicated within parentheses. The cells transfected with the FITC-conjugated CesA1-antisense ODN showed an increase of the mean FITC fluorescence intensity at T = 0 and T = 6 when compared to mock transfection controls, suggesting the antisense ODN could effectively enter the ecdysal cysts using the liposome-mediated oligonucleotide transfection method. [file Image_2.JPEG]

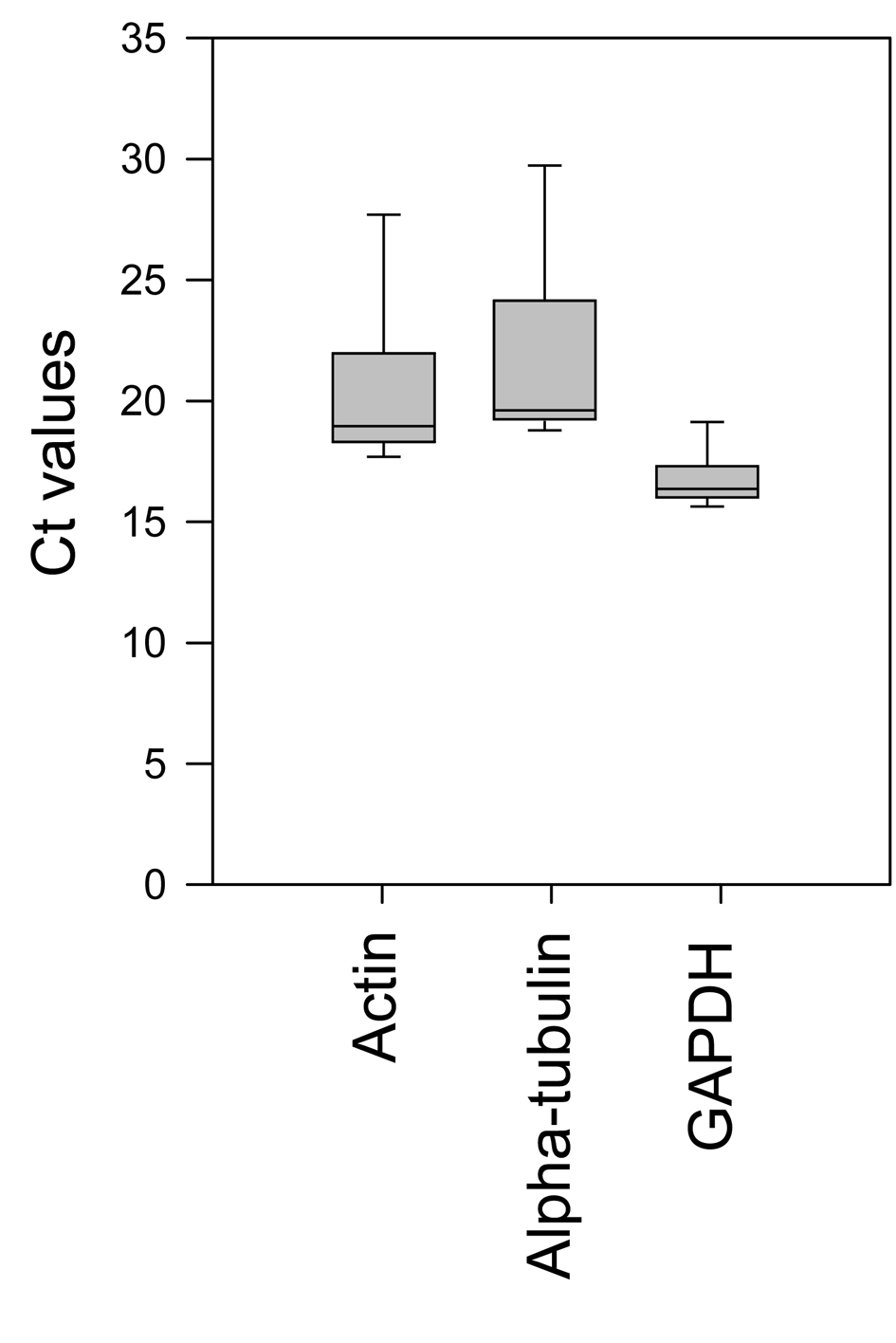

Supplement: FIGURE S3 — GAPDH was the most stable reference gene for quantitative real-time PCR. Box and whisker plots showing the Ct (cycle threshold) values of all tested reference genes. The horizontal line in the middle of each box indicates the median, while the top and bottom borders of the box mark the 75th and 25th percentiles, respectively. The whiskers above and below the box mark the minimum and maximum. Among several commonly used reference genes, the most abundant gene was GAPDH (cycle threshold (Ct) value: ∼15 to 19) and the least was alpha-tubulin (Ct value: ∼18 to 29). The expression stability of all the three candidate reference genes could also be exhibited by the coefficient of variance (CV) values. The CV values were: GAPDH (1.17%), actin (9.59%), and alpha-tubulin (19.34%). [file Image_3.JPEG]

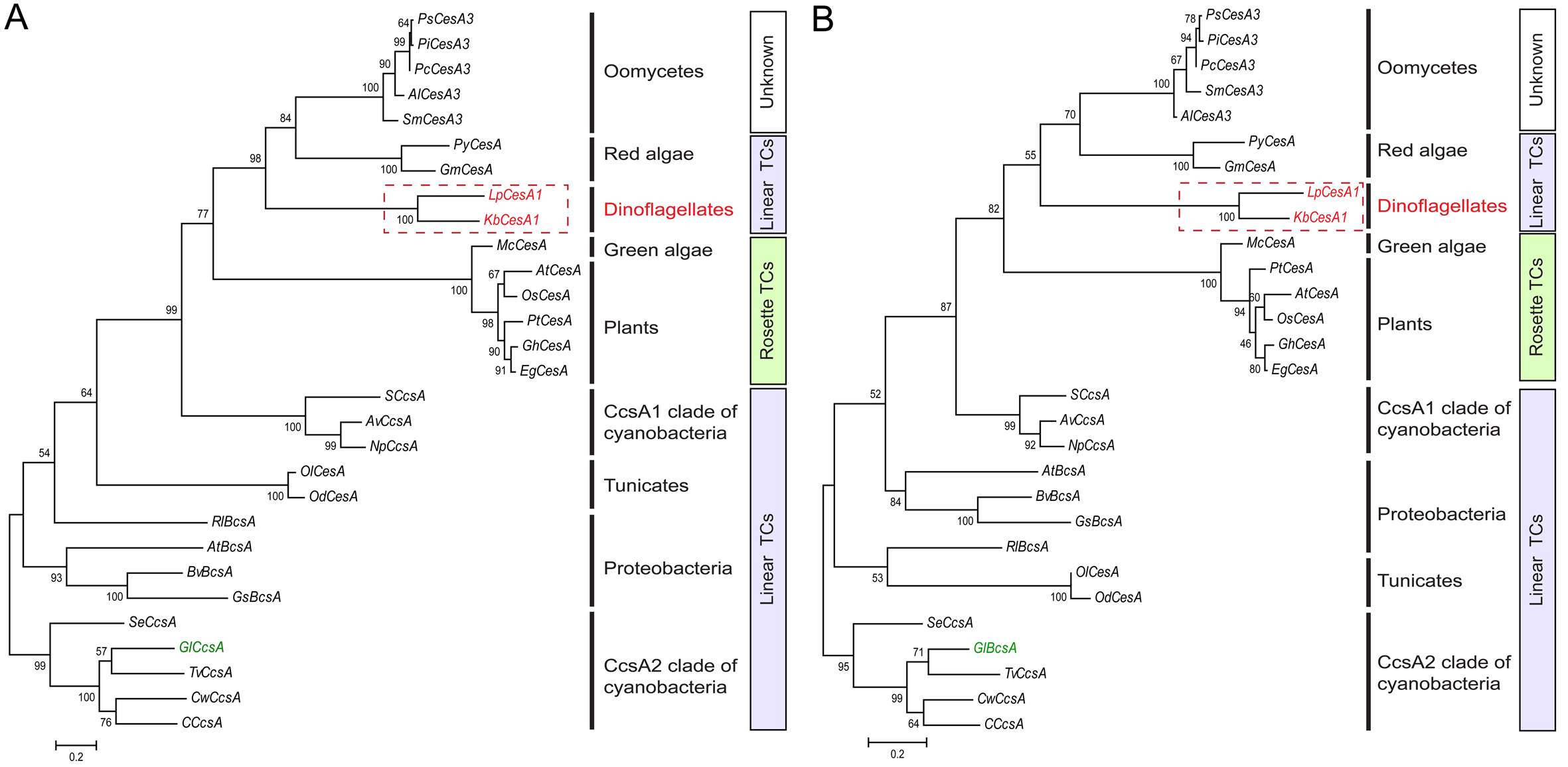

Supplement: FIGURE S4 — Phylogenetic trees constructed with representative CesA orthologs. Maximum-likelihood consensus trees [rooted with cyanobacterial CesAs (CcsA2 clade)] of (A) selected CesA orthologs based on full-length sequences and (B) conserved U1 to U4 regions. Both trees have the same topology. Dinoflagellate CesA1 orthologs share a node with other putative linear-type eukaryotic orthologs. Bootstrap values of 1,000 replicates are indicated at the nodes. GenBank accession numbers listed in Methods. Bar represents the phylogenetic distance of 0.2 amino acid substitution per site. [file Image_4.JPEG]

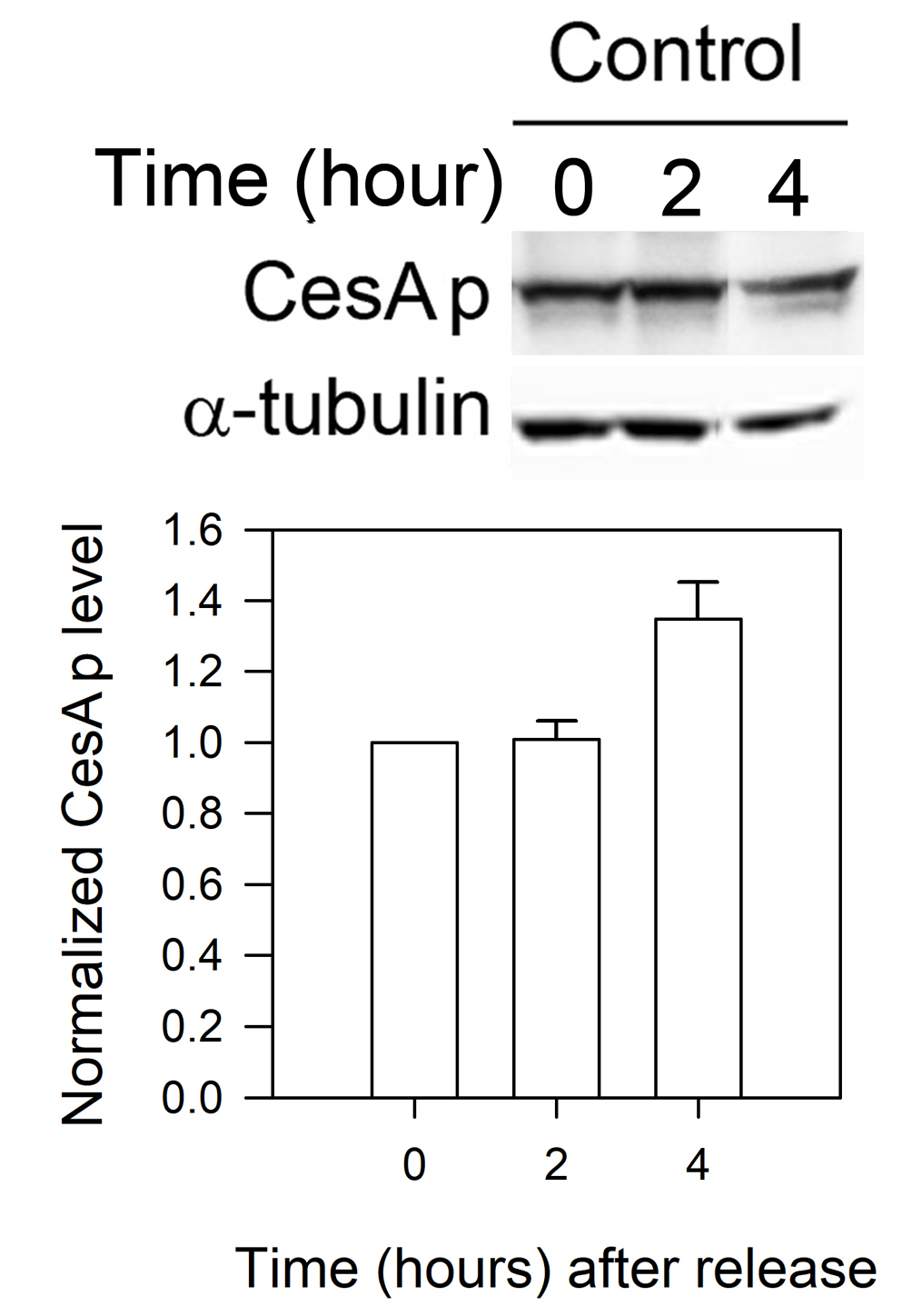

Supplement: FIGURE S5 — Expression of CesA1p during early cyst-to-swarmer transition. Anti-CesA1p immunoblot of cell lysates collected during early cyst-to-swarmer transition (TC–S). At T = 2, there was no significant increase in CesA1p signals when compared to the control at T = 0. Western blot signals were determined using ImageJ and normalized to the corresponding alpha-tubulin signals. Antigen-purified anti-CesA1p antibody was used throughout the experiments. Data represent means ± SE of triplicate experiments. [file Image_5.JPEG]
