# Supplementary material for: Knockdown of Dinoflagellate Cellulose Synthase CesA1 Resulted in Malformed Intracellular Cellulosic Thecal Plates and Severely Impeded Cyst-to-Swarmer Transition
Source: Front Microbiol. 2019 Mar 19;10:546. doi: 10.3389/fmicb.2019.00546 (PMC6433935; doi:10.3389/fmicb.2019.00546)
Supplement: TABLE S1 — Primers used for the cloning of KbCesA1 gene and qPCR. [file Table_1.DOC]

**Supplementary Table S1.** Primers used for the cloning of *KbCesA1* gene and qPCR.

| **Primer** | **Sequence (5’ to 3’)** | **Used for** |
| --- | --- | --- |
| CSL | CCGTAGCCATTTTGGCTCAAG | Cloning of 5’UTR of *KbCesA1* |
| KbCesA-R1 | CCAACAGAAGCAGATTCACTAAGG | Cloning of 5’UTR of *KbCesA1* |
| KbCesA-F2 | AGCAACGGTGTGGTCGAGGTCCT | Cloning the middle part of *KbCesA1* |
| KbCesA-R2 | TCCATCTTGGCTGCTTGCCCGTGA | Cloning the middle part of *KbCesA1* |
| KbCesA-F3 | TCACGGGCAAGCAGCCAAGATGGA | Cloning of 3’UTR of *KbCesA1* |
| Oligo-dT | GGCCACGCGTCGACTAGTACTTTTTTTTTTTTTTTTT | Cloning of 3’UTR of *KbCesA1* |
| KbCesA-F | ATGGAATTCTTTCAAAAGGAC | Cloning of full length coding sequence of *KbCesA1* |
| KbCesA-R | CTAGTCTGAAGAAGCCCCAATC | Cloning of full length coding sequence of *KbCesA1* |
|  |  |  |
| Lp_Actin-F | CAGTGGCATGTGCAAAG | qPCR (Actin of *L. polyedrum*) |
| Lp_Actin-R | GTCCCCGATGTAACTGTC | qPCR (Actin of *L. polyedrum*) |
| Lp_GAPDH-F | GGCTAAGAAGGATGGTTGAG | qPCR (GAPDH of *L. polyedrum*) |
| Lp_GAPDH-R | GTCTGCTAGCTTCATGTCTG | qPCR (GAPDH of *L. polyedrum*) |
| Lp_α-tubulin-F | CATCATAGGGCGTGTTTCTC | qPCR (α-tubulin of *L. polyedrum*) |
| Lp_α-tubulin-R | CACGCATGTTGACTCAAGGA | qPCR (α-tubulin of *L. polyedrum*) |
